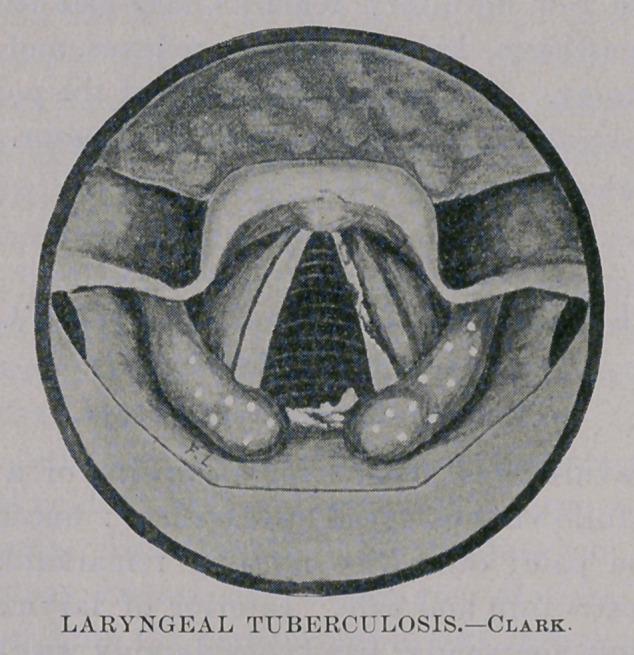# Laryngeal Tuberculosis—A Typical Case, with Illustration1From cases reported to the Section on Surgery, Buffalo Academy of Medicine, May 30, 1893.

**Published:** 1893-12

**Authors:** Horace Clark

**Affiliations:** 21 West North Street; Buffalo


### LARYNGEAL TUBERCULOSIS—A TYPICAL CASE WITH ILLUSTRATION.[1]

By HORACE CLARK, M. D., of Buffalo.

This photogravure[2] was made from a drawing of a throat which presented the following essential appearances : buccal and pharyngeal membrane pale; otherwise nothing remarkable about these parts. Epiglottis thin and pale. Interior of larynx pale ; secretion scanty ; left ventricular band considerably swollen, partially obscuring the vocal cord on that side ; several small yellowish points scattered along the free margin of the left cord ; also extensive ulceration upon this cord ; the right cord is thin and apparently free from disease. Both arytenoids are swollen ; the swelling is semi-opaque, and extends throughout the whole length of the ary-epiglottic ligaments ; symmetrically upon these ligaments are numerous yellowish white points ; there are two cone-shaped elevations of unequal size in the inter-arytenoidal space ; the smaller is in the median line and is truncated, with jagged upper edge, in comparison with the larger, which is intact and lies to the left ; confluent ulceration, of ill-defined, irregular outline, occupies the remainder of this space to the right.

1. From cases reported to the Section on Surgery, Buffalo Academy of Medicine, May 30, 1893.

2. Page 276.

The case is taken from my clinic records for May, 1893. Woman, thirty-one years old and married; four living, healthy children; one child died in infancy of consumption; clear hereditary and personal phthisical history. Three weeks previous to visit, throat felt as if it were "growing up;" at this time noticed pain in the throat, which was pretty constant, increasing daily, and aggravated upon swallowing.

There are phthisical cavities in the upper parts of both lungs.

This history, in conjunction with the result of physical examination, belongs with the largest figures in the statistics of the disease, with the exception of sex.

LARYNGEAL TUBERCULOSIS.—CLARK.

Three facts are herein illustrated:

1. Regional origin. Development and its bearing upon diagnosis from syphilis.

2. Difficult and painful swallowing as the most prominent symptom.

3. The best method of treatment.

1. The disease usually (*a*) begins in the posterior commissure, in front of the arytenoid cartilages, working its way outwards through the sub-mucosa, thence extending symmetrically to the ary-epiglottic folds. This *symmetrical* arrangement of the ulceration and tumefaction is a very important factor in the (*b*) differential diagnosis from syphilis. In syphilis the ulcers are most frequently *unilateral*, *single*, and *large;* in tuberculosis they are *bilateral*, *numerous*, and *small*; in syphilis the ulcers are surrounded by an *inflammatory zone;* in tuberculosis their *margin is*

*pale.* If the *epiglottis* is the seat of ulceration, syphilis seems to have a predilection for its *upper surface* and *free margin*; whereas the ulceration of tuberculosis is *lower down and at its base.* In syphilis the *thickening is irregular*; in tuberculosis it is *smoother and more uniform.*

2. It is such a picture as this, or one closely allied to it, which is most commonly seen when the phthisical patient comes for assistance with the first symptom which he is sure relates to his throat. This refers to *difficult and painful swallowing.* Heretofore there may have been a noticeable increase in the cough, and in the amount of expectoration, accompanied, perhaps, with an exacerbation in the general decline. All this the patient has related to his lungs. Difficult and even painful swallowing may very well occur before the epiglottis is involved.

The epiglottis is usually represented in the books as greatly swollen. Within two months from the date of the first visit, the epiglottis in this case would have measured half an inch across its upper border. More extensively ulcerated areas are also shown. Anyone familiar with such cases could not fail to make a diagnosis at any stage of the disease. The appearances shown in this picture could scarcely be mistaken by the learner.

3. It is at about this stage of tubercular laryngitis, when the best results are obtained from operative procedure for the relief of the distressing symptoms, or, indeed, for the arrest of the disease. Operation consists in the liberal use of the curette upon the ulcerated surfaces.[1] This is followed by the rubbing in of lactic acid, in solution varying in strength from 20 per cent. to 80 per cent., according to the choice of the operator. The result is the formation of cicatricial tissue.[2] Schmidt[3] devised a set of knives, for the purpose of incising the swollen parts, particularly the ary-epiglottic ligaments. Other observers find these enlargements to be hard, rather than soft and edematous. The knife does not give the result which would be expected from scoring a true edema. The explanation is, that an *appearance* of edema is caused, in part at any rate, by the infiltration of the membrane with tubercles.

It is stated that palliative treatment should always precede operation. The writer takes the view that, given such a case as the one here presented, no time should be lost in tentative efforts

1. The writer removes, in addition, such nodules as have not already broken down.
2. Hernyg: Die Heilbarkeit der Larynx-Phthise. Stuttgart, 1887, p. 57.
3. Cong. Internat. de Laryngol. Milan, 1880.

with palliative measures. Not that it is useless to rub in lactic acid alone,[1] but the formation of scar tissue is so much quicker and firmer when the parts have been first thoroughly scraped. Gratifying results are obtained by menthol used in the same way.[2] The writer's experience would justify the use of the curette in almost every case when ulceration can be made out; unless, indeed, the case has gone on to extremity. It is pretty generally laid down as a safe rule that, with extensive involvement of the epiglottis, instrumental interference is bad practice. In such cases palliative measures alone should be resorted to, including the hydrochlorate of cocaine in the form of a spray.

21 West North Street.

1. Krause: *Berlin Klin. Woch.*, 1885, Vol. XXII., p. 462.
2. Rosenberg: *Therap. Monatsheft*, 1888, Nos. 7 and 8. C. H. Knight: *Journal American Medical Association*, 1890, Vol. XIV., p. 89.
3. Read before the Buffalo Academy of Medicine, October 31, 1893.

## Figures and Tables

**Figure f1:**